# Novel Therapeutic Approach to Induce Autophagy in a *Drosophila* Model for Huntington’s Disease

**DOI:** 10.3390/cells9020495

**Published:** 2020-02-21

**Authors:** Marta Martinez-Vicente

**Affiliations:** Neurodegenerative Diseases Research Group, Vall d’Hebron Research Institute-Center for Networked Biomedical Research on Neurodegenerative Diseases (CIBERNED)-Autonomous University of Barcelona, 08035 Barcelona, Spain; marta.martinez@vhir.org

Autophagy induction is an attractive therapeutic approach to ameliorate aggregate accumulation in many neurodegenerative diseases. In Huntington’s disease (HD) in vivo models, a number of genetic and pharmacological mechanisms aimed to induce autophagy have been successfully tested [1], demonstrating the role of autophagy in promoting the elimination of mutant huntingtin (mHTT) aggregates and its neuroprotective effect. In their recent report in Cells, Vernizzi and colleagues [2] presented a totally new mechanism to induce autophagy, promote the elimination of mHTT aggregates, and ultimately achieve neuroprotection. This novel therapy is based on the overexpression of glutamine synthetase 1 (GS1), an enzyme that catalyzes the synthesis of L-glutamine from L-glutamate as part of the glutamate glutamine cycle (GGC), a physiological process between glia and neurons that controls glutamate homeostasis [3].

The GGC is often found compromised in neurodegenerative diseases including Huntington’s disease [4]. Previous studies have shown that GGC is impaired in mouse and *Drosophila* models of HD [5]. In postmortem brain samples from HD patients, GS1 levels were also reduced [6]. In this same context, the authors showed for the first time that human fibroblast derived from patients with HD also presented lower levels of GS1, confirming the alteration of the GGC in HD. Overall, the goal of the study was to study the contribution of GS1 to HD and investigate a new therapeutic approach based on the restoration of this enzyme in neurons. 

Authors have used a well-characterized in vivo model of HD; the Htt-Q93 model of *Drosophila* expresses the mutated human exon1 carrying and expanded polyglutamine (polyQ) tract with 93 glutamines and mimics many aspects of the neurodegeneration induced by toxic polyQ expansion in HD patients including neuronal loss, aggregate formation, and motor dysfunction [7]. This in vivo model is an extremely useful model to validate new therapeutic approaches, especially by genetic manipulation, since *Drosophila* models allow for the rapid generation of strains with over expression and downregulation of genes, and in addition, neurological biochemical changes and locomotion dysfunction can be easily achieved. 

Results presented by Vernezzi and colleagues showed that the expression of the *Drosophila* homolog of human GS1 in neurons ameliorates the phenotype induced by the expression of Htt-Q93 including a decrease in the locomotion defects, reduction of the Htt-Q93 aggregates in neurons, and reduction of neuronal death.

Interesting, the mechanism of action of the neuroprotective effect of GS1 overexpression was shown to be exerted through the activation of autophagy. Authors observed that GS1 expression could increase the autophagic flux in neurons (observed by the quantification of the *Drosophila* analogs of LC3 and p62/SQSTM1 proteins) and more importantly, the therapeutic effects were entirely autophagy-dependent, since the flies that lacked some of the essential genes for autophagy induction like *Atg5* and *Atg1* (ULK1 in mammals) could not rescue the Htt-Q93 phenotype after GS1 expression. 

How can the overexpression of an enzyme in the GGC induce autophagy? To answer this question, authors have analyzed the levels of glutamate, glutamine, and other essential and no-essential amino acids that are changed as a consequence of GS1 expression. The target of rapamycin (TOR) is the master regulator of autophagy and acts as a general sensor of the cellular nutrient and metabolic status. TOR can form two different multicomponent complexes, TOR complex I and TOR complex 2, both complexes are at the core of an intricate signaling pathway that can regulate diverse cellular functions such as autophagy, protein transcription, mRNA translation, cell growth, proliferation, ribosome biogenesis, mitochondria biogenesis, and cytoskeleton organization among others [8]. 

TORC1 negatively regulates autophagy induction by the phosphorylation of the ULK1 complex (Atg1 in *Drosophila*), thus inactivation of TORC1 can change the phosphorylation state of Atg1/ULK1, and allow the initiation of the autophagy machinery. Various cellular signals including amino acid concentration, but also ATP levels, growth factors, insulin, or hypoxia can inactivate TORC1. The authors found changes in different amino acid levels after GS1 expression, which could affect TORC1 activity. S6K protein is one of the most well characterized direct substrates of TORC1 and is used as a reporter of the activation state of the TOR. The authors found that the expression of GS1 in neurons significantly reduced the levels of S6K phosphorylation, suggesting the inactivation of TORC1 under these conditions. Accordingly, the authors proposed that GS1 expression in neurons changes the amino acid levels to mimic the metabolic condition (starvation-like) to inactivate TOR and trigger autophagy induction. 

This study uncovers a novel function for GS1 in Huntington’s disease and highlights the role of the GGC in neuronal homeostasis, opening a new therapeutic opportunity. Recovering GS1 expression is able to ameliorate neuronal survival by modulating the amino acid levels that initiate the TOR-dependent induction of autophagy.

Future works should now investigate if the autophagic induction described in this HD fly model through the expression of the GS1 enzyme could be a general therapeutic mechanism that could be reproduced in other neurodegenerative models with aggregate accumulation, and consequently if alternative pharmacologically treatments could reproduce the effect.
